# Ischemic Colitis in the Setting of Tirzepatide Use: A Potentially Underrecognized Adverse Effect

**DOI:** 10.7759/cureus.116973

**Published:** 2026-09-26

**Authors:** Isabel Jaszewski, Jean C Cruz Garcia, Karina Moreira, Ana E Suarez, Rebecca Cherner

**Affiliations:** 1 Family Medicine, Broward Health Medical Center, Fort Lauderdale, USA; 2 Family Medicine, Nova Southeastern University Dr. Kiran C. Patel College of Osteopathic Medicine, Fort Lauderdale, USA

**Keywords:** adverse drug effect, gastrointestinal tract complications, glucose-dependent insulinotropic polypeptide (gip)/glucagon-like peptide-1 (glp-1) receptor agonist, ischemic colitis, tirzepatide

## Abstract

Ischemic colitis is an uncommon but serious cause of acute abdominal pain that may result in bowel necrosis and may require surgical intervention. We report the case of a 63-year-old female patient with multiple comorbidities and a history of ischemic colitis, who developed sudden-onset severe cramping and abdominal pain, together with hypotension and diaphoresis, shortly after receiving her weekly tirzepatide injection. The patient reported suffering from chronic constipation with increased frequency of laxative use after beginning tirzepatide (Mounjaro). Laboratory evaluation demonstrated leukocytosis and lactic acidosis, while computed tomography (CT) findings were consistent with ischemic colitis. Her condition rapidly progressed to hypovolemic shock and acute kidney injury (AKI), requiring intensive care unit (ICU) admission and segmental bowel resection with colostomy creation. Surgical pathology report confirmed patchy mucosal necrosis consistent with ischemic colitis. This case raises concern for a potential association between tirzepatide use and ischemic colitis in a patient with preexisting risk factors. Clinicians should maintain a high index of suspicion for ischemic colitis in patients receiving tirzepatide who present with severe abdominal pain, particularly in the setting of worsening constipation or a prior history of ischemic colitis. This case raises the question of whether a history of ischemic colitis and/or chronic constipation may warrant clinical caution and pharmacovigilance before starting these medications.

## Introduction

Glucagon-like peptide-1 receptor agonists (GLP-1 RAs) are a class of medications that mimic endogenous incretin hormone activity, enhancing glucose-dependent insulin secretion, suppressing glucagon release, delaying gastric emptying, and increasing satiety [1]. These effects improve glycemic control and promote weight loss, contributing to their widespread use in type 2 diabetes and obesity management [1-3]. However, gastrointestinal (GI) adverse effects such as nausea, vomiting, diarrhea, and constipation are commonly reported [1,2,4].

Ischemic colitis is an uncommon but potentially serious condition [5-7] resulting from reduced colonic blood flow due to hypoperfusion, vascular occlusion, or vasospasm, often affecting watershed areas such as the splenic flexure [7]. Clinically, patients typically present with acute lower abdominal pain, hematochezia, and urgency. Key predisposing risk factors include baseline GI motility disorders, particularly chronic constipation, as well as cardiovascular disease via structural plaque or decreased perfusion pressure, metabolic comorbidities, and a prior history of ischemic colitis, which signals heightened microvascular susceptibility. While typically reversible when perfusion is restored, severe or prolonged ischemia can progress to necrotic colitis with transmural infarction requiring surgical resection. Most cases are nonocclusive, driven by systemic hypoperfusion rather than primary arterial obstruction.

Although GLP-1 RAs have theoretical mechanisms that could contribute to hypoperfusion (e.g., delayed GI transit and volume depletion), large meta-analyses have not demonstrated a significant increase in GI ischemic events, often citing cholelithiasis, gastroesophageal reflux disease (GERD), and gastroparesis as statistically significant [1,3], and some preclinical data suggest potential intestinal protective effects. Nevertheless, rare case reports have described possible temporal associations with intestinal ischemia [5-7].

## Case presentation

We present a 63-year-old female patient with a history of type 2 diabetes mellitus, hyperlipidemia, chronic constipation, chronic obstructive pulmonary disease (COPD), arthritis, and prior ischemic colitis status post bowel resection, who presented to the emergency department with acute abdominal pain and intermittent abdominal cramps that began earlier that same morning while she was en route to board a cruise ship. She reported that she typically experiences cramping with bowel movements, which she attributed to her chronic laxative use; however, she described that day's pain as qualitatively different and more severe. The patient’s partner noted that she became diaphoretic, fatigued, and had sudden poor responsiveness, prompting Emergency Medical Services (EMS) transport to the hospital. The patient denied nausea, vomiting, fever, chills, or changes in bowel habits. Notably, she had injected her weekly tirzepatide that same morning, approximately 2-3 hours before symptom onset. This patient had been using tirzepatide for six months at the time of presentation.

On presentation to the emergency department, she was hypotensive with a blood pressure of 71/31 mmHg, a pulse of 78 bpm, a respiratory rate of 19, a temperature of 36.3°C, and an oxygen saturation of 98% on room air. Laboratory evaluation revealed metabolic acidosis with an elevated lactic acid of 2.2 mmol/L, a creatinine of 1.5 mg/dL with a baseline of 0.7-0.9 mg/dL, a mildly elevated aspartate aminotransferase (AST) of 40 U/L, a leukocytosis of 18.5 × 10³/μL with a left shift, and a thyroid-stimulating hormone (TSH) value of 5.73 mIU/L (Table 1). An abdominal X-ray demonstrated a normal nonobstructive bowel gas pattern. A computed tomography (CT) of the abdomen and pelvis without intravenous contrast revealed a diffusely distended, fluid-filled colon with several areas of mesenteric induration (Figure 1), raising concern for ischemic colitis. There was no evidence of pneumatosis or portal venous gas.

**Table 1:** Patient lab values collected in the emergency department on initial presentation

Lab test	Initial patient values	Reference range
Lactic acid	2.2 mmol//L	0.5-2.2 mmol/L
Creatinine	1.5 mg/dL (baseline: 0.7-0.9 mg/dL)	0.7-1.3 mg/dL
Aspartate aminotransferase (AST)	40 U/L	5-34 U/L
White blood cell count (WBC)	18.5 x 10^3^ cells/µL	4.0-11.0 x 10^3^ cells/µL
Thyroid-stimulating hormone (TSH)	5.73 mlU/L	0.4-4.5 mlU/L

**Figure 1:**
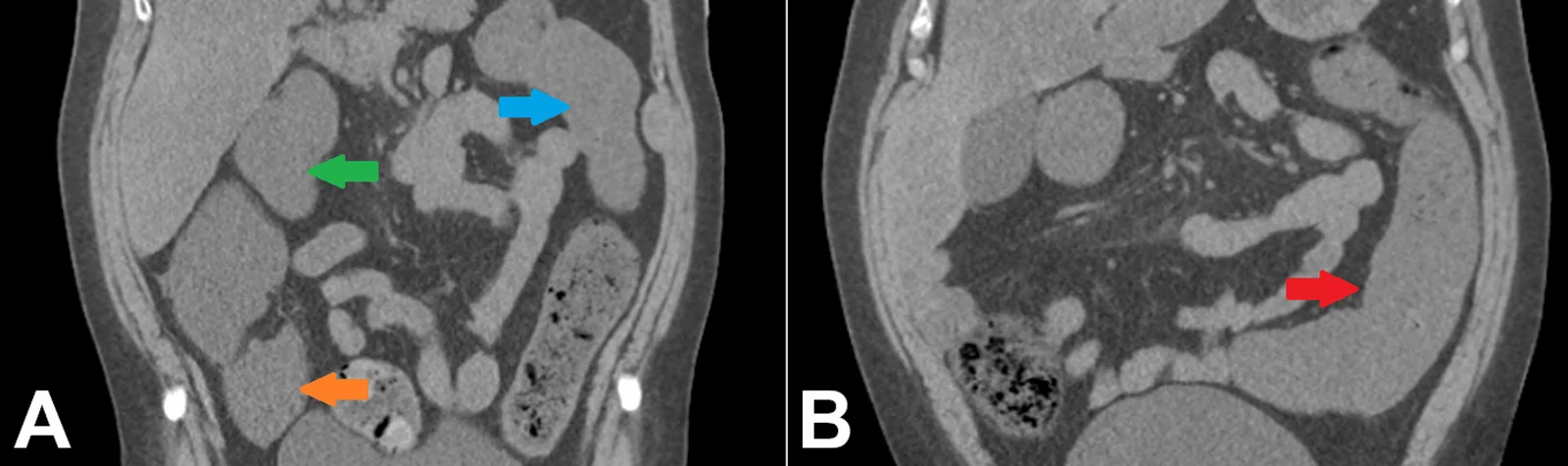
Computed tomography of the abdomen and pelvis demonstrating significant colonic distension in the cecum, ascending, descending, and sigmoid colon with mesenteric induration in the anterior-posterior view (A) Distension of the cecum (orange arrow), ascending (green arrow), and descending (blue arrow) colon. (B) Distension of the sigmoid colon (red arrow)

On examination, the patient was alert and oriented but in severe acute distress due to pain. Abdominal examination revealed generalized distension and severe tenderness to palpation, particularly in the left upper and lower quadrants. A nasogastric tube (NGT) was placed and yielded a brown output. She was admitted to the intensive care unit (ICU) for vasopressor support with norepinephrine, broad-spectrum antibiotics (piperacillin/tazobactam), bowel rest, and close monitoring of labs, including lactic acid and renal function. General surgery was consulted due to concern for ischemic bowel, and bowel prep with GoLYTELY was initiated as tolerated due to concern for constipation being the initial cause of the patient’s symptoms.

The following morning, the patient's clinical status was not improving, and she subsequently underwent open laparotomy with segmental bowel resection and colostomy creation. Pathology demonstrated patchy mucosal necrosis consistent with ischemic colitis. Her hospital course was complicated by a transient setback due to hypotension, rising leukocytosis, nausea, vomiting, and worsening abdominal tenderness. Repeat CT imaging and clinical evaluation revealed a small bowel obstruction (Figure 2). This was managed with repeat insertion of an NGT, nothing by mouth (NPO) designation, and fluid resuscitation. The patient was able to decompress successfully, leading to the removal of her NGT and advancement of the diet. She was ultimately cleared for discharge with close follow-up planned with gastroenterology, her primary care physician, and general surgery.

**Figure 2:**
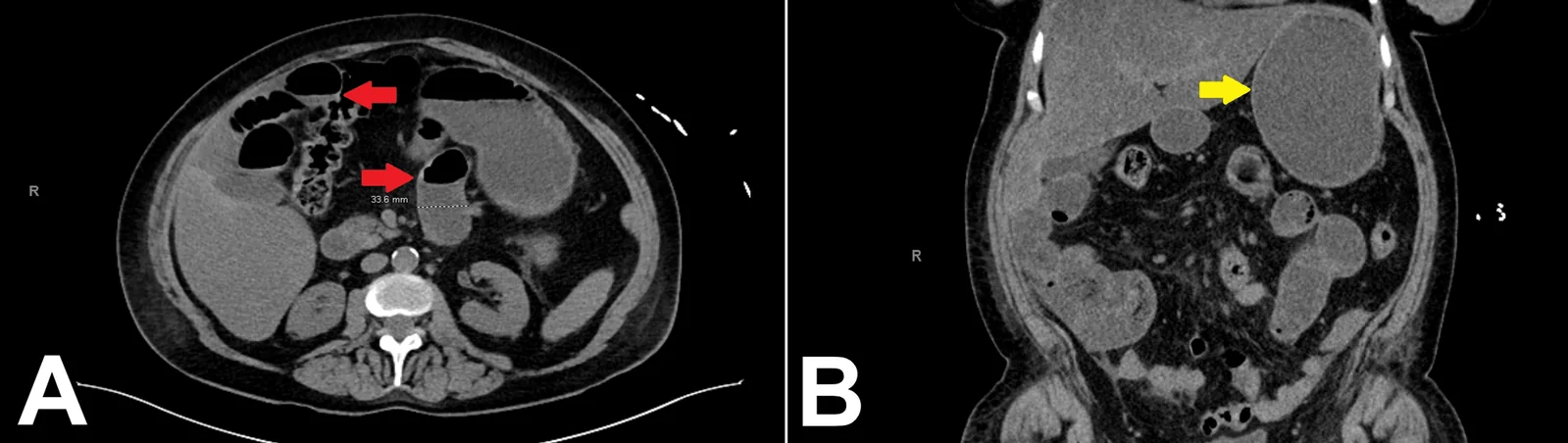
Repeat CT scan of the abdomen and pelvis (axial and coronal view) demonstrating dilation of small bowel with air fluid levels. The small bowel measured up to 3.36 cm. Gastric distention is also present. Findings suggestive of partial small bowel obstruction (A) Dilation of the small bowel with air-fluid levels (red arrows). (B) Distension of the stomach (yellow arrow)

This case raises concern for a possible association between tirzepatide and ischemic colitis, possibly mediated by worsening constipation, delayed GI transit, and colonic hypoperfusion. It underlines the importance of early evaluation and consideration of ischemic colitis in patients receiving GLP-1 RAs and presenting with acute, severe abdominal pain. It also raises the concern that a history of prior ischemic colitis may represent a relative contraindication to initiating these medications.

## Discussion

Tirzepatide, a dual glucose-dependent insulinotropic polypeptide (GIP)/GLP-1 receptor agonist, may have contributed to ischemic colitis in this patient by slowing gastric emptying and intestinal transit, leading to constipation, increased intraluminal pressure, and impaired mucosal perfusion [5-7], especially in those with baseline GI motility issues (Figure 3). 

**Figure 3:**
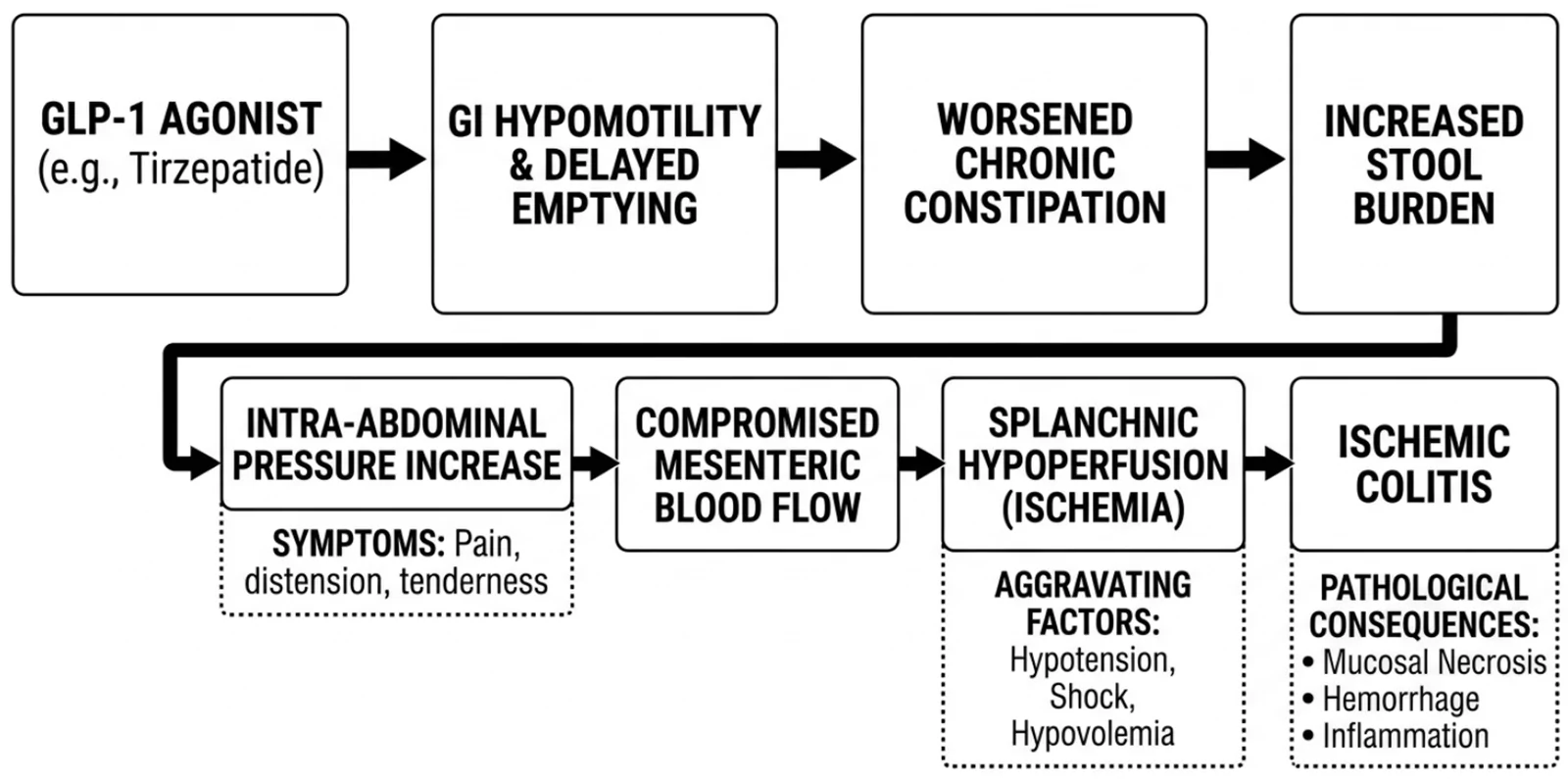
Proposed pathophysiologic mechanism of GLP-1 agonist-induced ischemic colitis GLP-1: glucagon-like peptide-1 This flowchart illustrates how GLP-1 receptor agonist therapy can contribute to gastrointestinal hypomotility, increased intra-abdominal pressure, and compromised splanchnic perfusion, ultimately leading to colonic ischemia

In this patient, the severe presentation was potentially driven by a convergence of multiple key risk factors. Baseline chronic constipation is a known risk factor for ischemic colitis; fecal stasis can worsen localized hypoperfusion by increasing intraluminal pressure and impairing venous outflow. Additionally, underlying metabolic comorbidities, specifically type 2 diabetes mellitus, can promote microvascular dysfunction and impair colonic arterial autoregulation. In this patient, chronic constipation, laxative use, and long-standing diabetic microvascular susceptibility likely created a baseline vulnerability that was acutely exacerbated following tirzepatide exposure [6]. 

Ischemic colitis results from segmental hypoperfusion, most commonly affecting watershed areas like the splenic flexure and sigmoid colon, and can progress to transmural necrosis [8]. In this case, the clinical course was further compounded by severe systemic hypotension requiring ICU care for hypovolemic shock and acute kidney injury (AKI), which severely compromised splanchnic perfusion pressure. It is important to note that this is the second occurrence of ischemic colitis in this patient, demonstrating a pre-existing vascular vulnerability, as well as her second time undergoing bowel resection with colostomy creation.

A prior history of ischemic colitis raises concern for recurrence with GLP-1/GIP therapy. When superimposed on acute hypotension, chronic constipation, and diabetic microvasculopathy, GLP-1/GIP receptor agonists may precipitate severe colonic ischemia. Although causality is not established, with a Naranjo score of 7 (Table 2), these agents should be used cautiously in higher-risk patients (e.g., prior ischemia, severe constipation, bowel obstruction, or significant GI surgical history) [5,6,9]. Clinicians should maintain high suspicion for ischemic colitis in patients on GLP-1 therapy presenting with acute abdominal pain, particularly when constipation or dysmotility is present.

**Table 2:** Naranjo Adverse Drug Reaction Probability Scale ≥9 points: The reaction is almost certainly due to the medication. 5-8 points: The reaction is likely due to the medication. 1-4 points: The reaction could be related to the medication, but other factors are more likely to be the cause. ≤0 point: The reaction is unlikely to be related to the medication Source: [9]

Question	Yes	No	Do not know	Score
Are there previous conclusive reports on this reaction?	x			1
Did the adverse event appear after the suspected drug was administered?	x			2
Did the adverse event improve when the drug was discontinued or a specific antagonist was administered?	x			1
Did the adverse event reappear when the drug was readministered?			x	0
Are there alternative causes that could on their own have caused the reaction?			x	0
Did the reaction reappear when a placebo was given?		x		1
Was the drug detected in blood or other fluids in concentrations known to be toxic?		x		0
Was the reaction more severe when the dose was increased or less severe when the dose was decreased?	x			1
Did the patient have a similar reaction to the same or similar drugs in any previous exposure?		x		0
Was the adverse event confirmed by any objective evidence?	x			1
Total				7

## Conclusions

Currently, there is little existing literature addressing the relationship between GLP-1 receptor agonists and rare but serious GI side effects, such as ischemic colitis. Although constipation, pancreatitis, and colitis are occasionally cited as rare adverse effects of GLP-1 receptor agonists, their connection to GLP-1 use may be underrecognized in clinical settings. Chronic constipation represents a modifiable risk factor that may exacerbate ischemic injury in predisposed patients. Given that patchy mucosal necrosis is a hallmark of moderate disease, early recognition and prompt supportive management are essential, with timely surgical intervention required in severe or complicated cases. Clinicians must carefully review GLP-1/GIP receptor agonist prescriptions in patients with significant baseline GI risk factors and thoroughly counsel patients on potential severe adverse effects. Clinicians should maintain a high suspicion for ischemic colitis in patients receiving GLP-1/GIP receptor agonists who present with acute abdominal symptoms, and address medication discontinuation if indicated. Further investigation is warranted to better define whether factors such as prior ischemic colitis, extensive abdominal surgery, bowel obstruction history, and chronic constipation should influence pre-treatment screening and heightened clinical vigilance, patient counseling regarding warning signs of severe dysmotility/hypoperfusion, and proactive constipation management when initiating these therapies.
